# The correct diagnosis and therapeutic management of tear dysfunction: recommendations of the P.I.C.A.S.S.O. board

**DOI:** 10.1007/s10792-017-0524-4

**Published:** 2017-04-10

**Authors:** Maurizio Rolando, Emilia Cantera, Rita Mencucci, Pierangela Rubino, Pasquale Aragona

**Affiliations:** 1 IsPre Oftalmica, Ocular Surface Center, Genoa, Italy; 2 Israelitic Hospital, Rome, Italy; 3 Eye Clinic, University of Firenze, Florence, Italy; 4 Eye Clinic, Azienda Ospedaliero-Universitaria di Parma, Parma, Italy; 5 Eye Clinic, Department of Biomedical Sciences, University of Messina, Messina, Italy

**Keywords:** Tear dysfunction, Ocular surface, Diagnosis, Treatment

## Abstract

**Purpose:**

To describe a standard approach to manage tear dysfunction (TD), in order to obtain a clinically favourable outcome. TD is a highly prevalent, yet largely underdiagnosed, condition that affects from 5 to 30% of the population above 50 years old. Left untreated, TD is associated with eye discomfort and ocular surface disease, substantially affecting quality of life. Although the prevalence of this problem is increasing significantly, a standard approach to its prevention and treatment is not available yet.

**Methods:**

In September 2015, a team of Ocular Surface Italian Experts convened for a roundtable to discuss on the latest knowledge about diagnosis and treatments for TD and the real issues in the management of these patients. The discussion centred on the appropriate definition of TD, proposing a new classification of risk factors and how to identify them, how to make a correct diagnosis choosing the rational therapy (questionnaires, symptoms’ time relation, seasonality, low tech diagnostic manoeuvres, specific tests for the detection of tear film disturbances leading to recognition of the level of disease and of the ocular system elements involved), which artificial tear matches the ideal profile for a rational therapy and which questions should be done to the patient.

**Results:**

A multi-item flowchart for tear film dysfunction, with point-by-point explanatory guide, to better identify and manage the patient with this disorder is provided.

**Conclusions:**

The growing prevalence of TD demands increased attention. An appropriate prevention and a treatment pattern for the patient, combined with greater patient–practitioner interaction, and patient education is offered.

## The current understanding of tear dysfunction

Tear dysfunction (TD) is a disease frequently encountered in the clinical practice, characterised by an impairment of the ocular surface. The prevalence ranges between 5 and 33% of the world’s adult population [1, 2]. It is a chronic and progressive condition whose symptoms vary greatly from virtually none to invalidating.

The continuously growing number of patients who are referred from general ophthalmology outpatient clinics to specialist centres confirms this epidemiological finding.

It is a multifactorial disease that affects one or more elements of the ocular surface functional unit, which includes tear film, cornea, limbus, conjunctiva, lid margin muco-epidermal junction, and lacrimal gland tubulo-acinar epithelia, as well as lacrimal drainage system, and eyelids. The epithelia have an intense and continuous glycoprotein secretory activity, which maintains excellent relations with the lacrimal fluid. Moreover, they mutually affect each other in a simultaneous response for the maximal functional efficacy of the system [2–5].

The system also includes the eyelids and the nasolacrimal duct and recognises the supplementary functions of the immune, vascular, nervous and endocrine systems, which are useful for the refractive and protective properties of the tear film.

A sufficient amount of tears, a stable and even composition and architecture of the tear film, proper eyelid closure with normal blinking and suitable tear film turnover are the prerequisites for maintaining the homoeostasis of the ocular surface. Whatever the cause, the absence of these conditions is behind the Tear Dysfunction Syndrome (or Dry Eye Disease) [5].

**Fig. 1:**
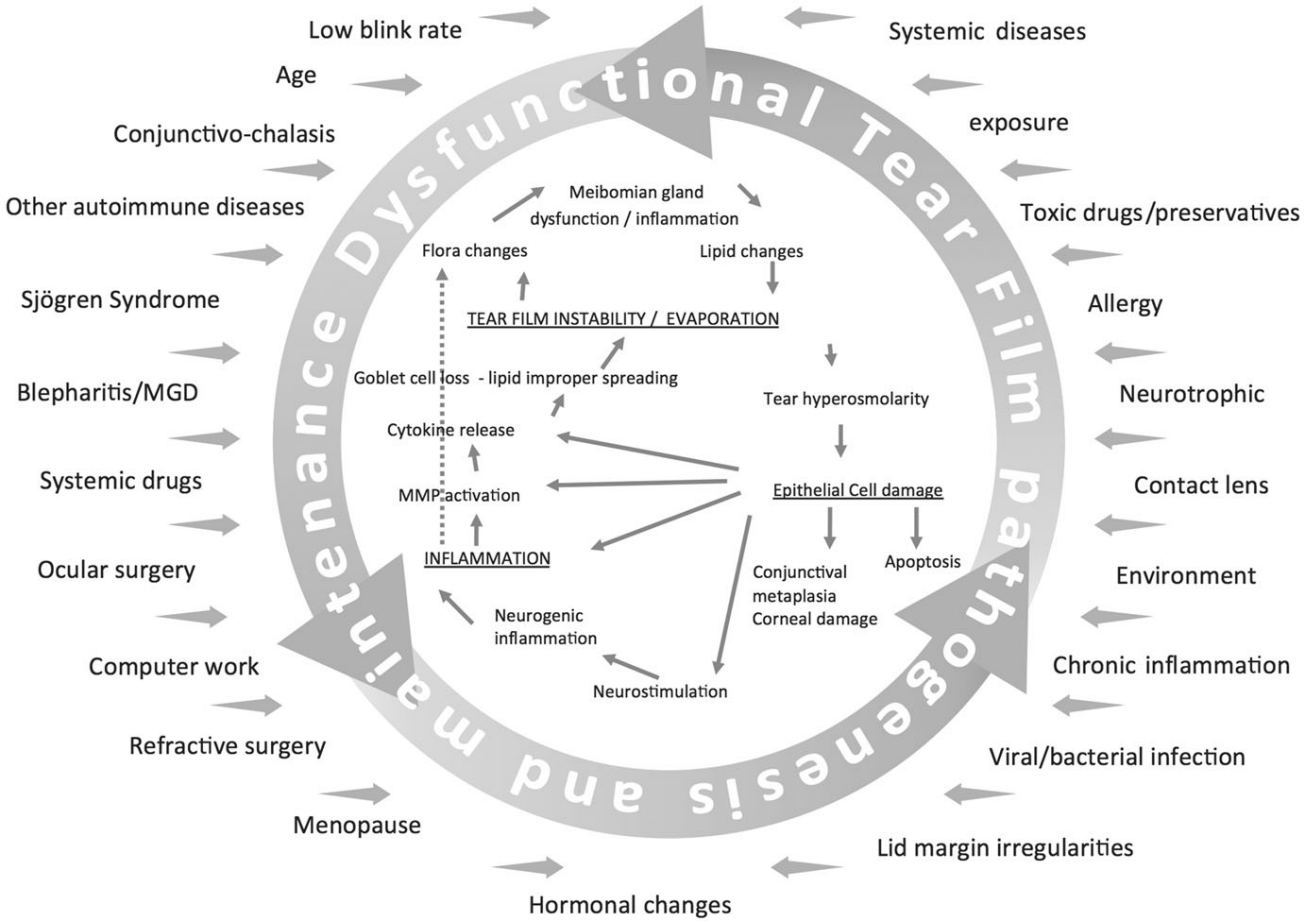
Dysfunctional tear film pathogenesis and maintenance. *MGD* meibomian gland dysfunction, *MMP* matrix metalloproteinase Adapted from DEWS 2007

If the ocular surface cannot adapt and quickly correct this “malfunction”, it enters a vicious circle, which leads to chronic damage and, subsequently, to the disease (Fig. 1).

**Fig. 2:**
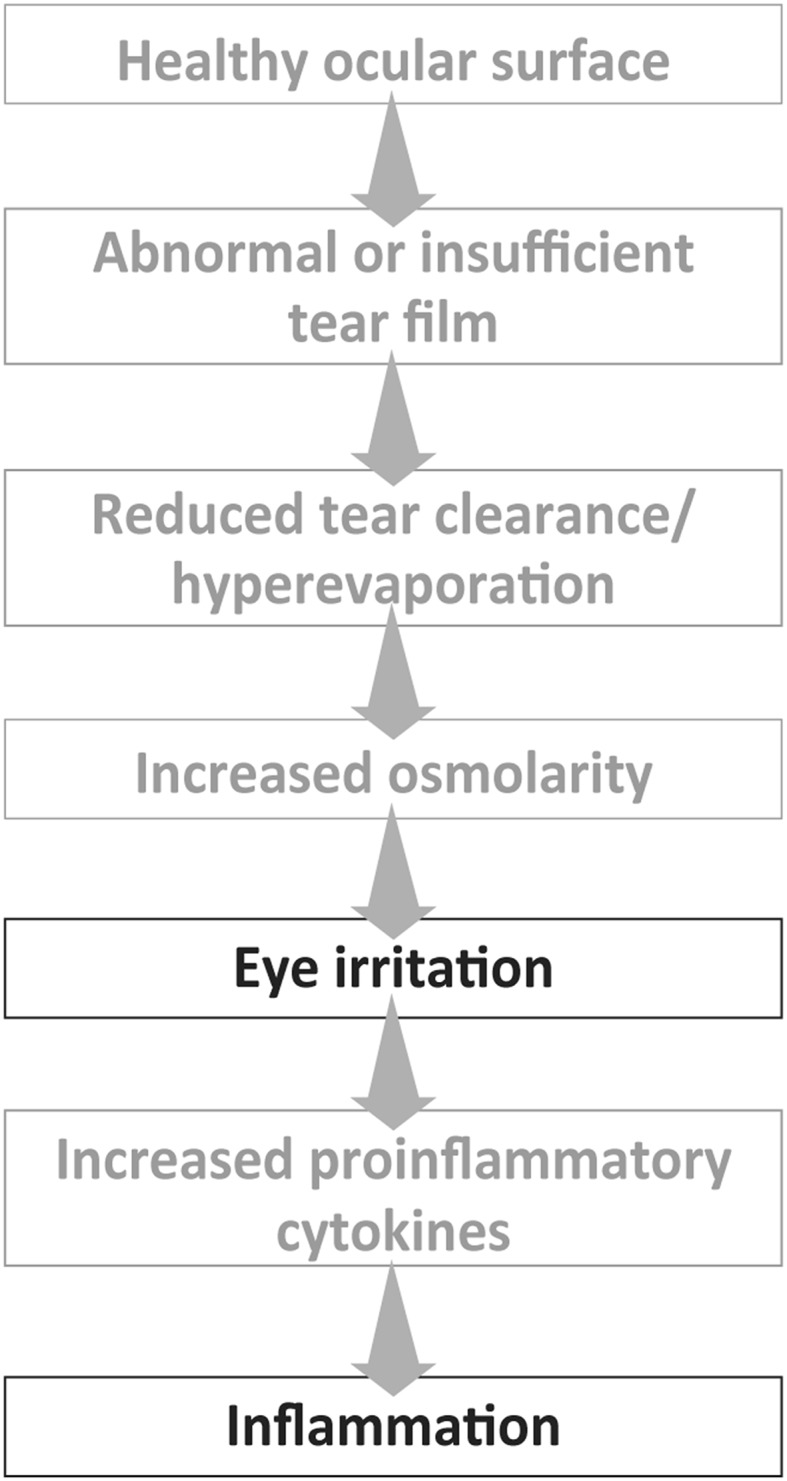
Inflammatory cascade Adapted from Rolando M. et al. Br J Ophthalmol 2010; 94 suppl l: l–9

As all patients present with chronic ocular surface disorders, patients with TD always have the typical signs of system failure including tear instability, epithelial suffering, and inflammation (Fig. 2).

Tear instability, or altered tear composition, drives the pathogenic process and is always associated with subclinical or clinically evident inflammation. Tear film instability can be caused by excessive evaporation, which results in an increased concentration of electrolytes. Hypertonicity in the epithelial environment involves the onset of inflammation and tissue damage [5–7].

Other causes of tear instability are primary tear hyposecretion (due to direct malfunction of the main glands) and secondary tear hyposecretion (e.g. prolonged inflammation resulting in reduced efficiency of the corneal nervous system, or anterior segment surgery). In these cases, inflammation is a consequence of the reduced tear clearance, which results in a reduced supply of epithelial growth and regulation factors and an increased in situ permanence of toxic factors that come from epithelial metabolism or the surrounding environment. Over time, the inflammation involves also the corneal nerve fibres and the eyelid glands, thereby triggering and maintaining the multiple pathogenic vicious circles, which characterise this disease. It is important to stress that the underlying inflammation is a key element, regardless of the factors that have caused it [8–11].

Tear Dysfunction Syndrome (or Dry Eye Disease) is a complex disease, which tends to sustain itself through the creation of vicious circles. As with all ocular surface disorders, three pathogenic factors are always present with different levels of expression according to the clinical presentations and level of the disease. These factors are: (1) tear instability, (2) epithelial malfunction and/or suffering and (3) more or less clinically evident inflammation [12, 13].

In summary, we can state that TD is part of the discomfort of the eye surface. The common name “dry eye” is linked to the concept of lack of tears. However, it is important to stress that the problem is that tears do not function because the ocular surface is not functioning.

## Medical history and local and general risk factors: identification and classification

Patients with TD often report eye conditions such as photophobia, foreign body sensation (or rather, in the words of the patients, “sand in eye feeling”), burning, itchiness, dryness, eye fatigue and pain. Patients can also develop redness, contact lens intolerance and, in some cases, mucus secretion. Most patients report a series of symptoms and not just one. It is interesting to stress that lacrimation is also a reported symptom, although it is mainly present in the early stages of the disease. Excess lacrimation is to be considered a paradoxical lacrimal reflex, since basal tear secretion is reduced in the event of dry eye [2].

The above-described symptoms are universally accepted by the scientific community. On the other hand, several other symptoms can be generically referred to as eye discomfort and can be indicators of TD [14, 15]. However, the medical history must always start with the identification of general and local risk factors, which can increase or predispose to a higher risk of ocular surface discomfort.

Risk factors can be general or local (Tables 1, 2).

**Table 1:** The three main pathogenic factors of the lacrimal dysfunction

Tear film instability			Epithelial suffering		Inflammation	
Reduced tear volume	Poor clearance	Changes in rheology and viscoelastic activity	Changes in the secretory activity, altered mucus glycoproteins	Reduced cell mobility and adhesion, apoptosis	Epithelial damage	Reduced tear secretion, reduced clearance
Direct epithelial trauma	Hyperosmolarity. Increased presence of inflammatory cytokines	Discomfort when blinking. Foreign body sensation	Tear instability, vision disturbances, blinking disorders	Punctate keratitis, pain, photophobia, vision disturbances, inflammatory stimulus	Keratitis and conjunctivitis, pain, alteration and loss of muciparous cells	Maintenance of inflammation, recruitment of antigen-presenting cells, shift to adaptive immune reactions

**Table 2:** Risk factors classification

General	Local
Advanced age	Surgery (refractive, cataract)
Gender (higher incidence in women)	Chronic therapies (glaucoma, vasoconstrictors)
Long-term and specific pharmaceutical therapies	Use of drugs with preservatives
Hormonal disorders	Blepharitis
Haematopoietic stem cell transplantation	Recent infections
Diabetes	Use of eye drops
Chemotherapy	Eye allergies
Environment	Use of contact lenses
Professional activity	
Autoimmune diseases	
Lifestyle (smoking, drinking, balanced diet)	
Dermatitis	
Menopause	
Allergies	
Pregnancy	
Hypovitaminosis A	
Hepatitis C	

A number of considerations about risk factors for Tear Dysfunction Syndrome (or Dry Eye Disease) should be raised:

### Why advanced age?

Tear production decreases with age due to the progressive involution of the lacrimal glands and nerve activities that regulate them. The continuous and consistent decrease in basal tear secretion and the resulting eye irritation often lead to an excessive production of reflex tears [2].

### Why are women more exposed?

In women aged between 40 and 60 years, the secreting portion of lacrimal glands undergoes progressive atrophy due to the new hormonal balance induced by menopause. Moreover, after menopause, androgen/oestrogen imbalance leads to the loss of control over inflammation typical of the androgenic component. Oestrogenic activity, which facilitates inflammation, can occur more easily [2, 16].

### Why the environment?

High altitude with increased UV exposure, sunny, dry, or windy weather conditions, environments with operating heating or air conditioning systems, increase tear evaporation and oxidative stress, thereby reducing eye lubrication [2].

### What are the risks of those who wear contact lenses?

Their use can increase tear evaporation significantly, causing irritation and infections. Corneal lens disinfecting and lubricating solutions can alter the lacrimal gland and tear production. Moreover, if the eye is not lubricated enough, the lens tends to adhere to the cornea, causing damage, which can be serious (abrasion, keratitis) [2, 17].

### Are there drugs that increase the risks of developing tear dysfunction?

Some drugs (hormones, immunosuppressants, decongestants, antihistamines, diuretics, antidepressants, beta blockers, heart disease- and ulcer-treating drugs) can inhibit the production of efficient tears [2].

### The impact of drugs and polypharmacy

With age, the number of drugs administered systemically increases. As we have seen, many of these drugs can interfere with tear production (Tables 3, 4).

**Table 3:** Classes of systemic medications that can induce dry eye and dry mouth

Class of drugs	
Anaesthesia adjuvants	Antipyretics
Analgesics	Antirheumatics
Antiandrogens	Spasmolytics
Antiarrhythmics	Antiviral drugs
Anticholinergic drugs	Anxiolytics
Antidepressants	Bronchodilators
Antiemetics	Chelating agents
Antihistamines	Decongestants
Antihypertensives	Diuretics
Antileprotics	Neurotoxins
Antimalarials	Opioids
Antimuscarinics	Psychedelic agents
Antineoplastics	Retinoids
Antiparkinson drugs	Sedatives–hypnotics
Antipsychotics

**Table 4:** Systemic drugs that may induce or worsen dry eye http://www.eyedrugregistry.com/

Class	Examples
Antihypertensives (beta-agonists)	Acebutolol
Antihypertensives (alpha agonists)	Atenolol
Antiarrhythmics (beta blockers)	Carvedilol
	Labetalol
	Metoprolol
	Corgard
	Pindolol
	Clonidine
	Prazosin
	Oxprenolol
	Propranolol
Antipsychotics	Chlorpromazine
	Fluphenazine
	Lithium carbonate
	Perphenazine
	Prochlorperazine
	Promethazine
	Quetiapine
	Thiethylperazine
	Thioridazine
	Brompheniramine
	Carbinoxamine
	Chlorphenamine (chlorpheniramine)
	Clemastine
	Cyproheptadine
	Dexchlorpheniramine
Bronchodilators	Diphenhydramine
Antispasmodics/antimuscarinics	Doxylamine
Antiarrhythmics	Ipratropium bromide
	Atropine
	Homatropine
	Tolterodine
	Hyoscine (scopolamine)
	Hyoscine methobromide (methscopolamine)
	Disopyramide
Antineoplastics	Busulfan
	Cyclophosphamide
	Interferon (alpha, beta, gamma, or PEG)
	Vinblastine
	Cetuximab
	Erlotinib
	Gefitinib
Antihistamines	Cetirizine
	Desloratadine
	Fexofenadine
	Loratadine
	Olopatadine
	Tripelennamine
Antidepressants	Citalopram
	Fluoxetine
	Fluvoxamine
	Paroxetine
	Sertraline
Antileprotics	Clofazimine
Antirheumatics/analgesics	Aspirin
	Ibuprofen
Sedatives–hypnotics	Primidone
Drugs secreted in tears	Aspirin
	Chloroquine
	Clofazimine
	Docetaxel
	Ethanol
	Hydroxychloroquine
	Ibuprofen
	Isotretinoin
Antiandrogens	Tamsulosin
	Terazosin
	Doxazosin
	Alfuzosin
Neurotoxins	Botulinum toxin A or B
Antimalarial agents	Chloroquine
	Hydroxychloroquine
Retinoids	Isotretinoin
Antiviral drugs	Aciclovir
Thiazides	Bendroflumethiazide
	Chlorothiazide
	Chlorthalidone
	Hydrochlorothiazide
	Hydroflumethiazide
	Indapamide
	Methyclothiazide
	Metolazone
	Polythiazide
	Trichlormethiazide
Cannabinoids	Dronabinol
	Hashish
	Cannabis
Chelating agents	Methoxsalen
Strong analgesics	Morphine
	Opium/opioids
Antipsychotic agents	Pimozide

A fundamental step in taking a medical history is to try to know which drugs have been administered systemically, because every agent that can interfere with one or more tear film components (lipids, water, mucins) can disrupt the stability of the tear film and cause or worsen the typical dry eye symptoms. Physician’s Desk Reference reports that 56% of the top 100 drugs sold in the USA in 2009 can cause eye dryness and 22% has a proven negative effect on tear secretion [18].

With age, there is a significant reduction in tear secretion associated with an increase in the number of systemic medications. After a while of taking drugs for other intercurrent diseases, these patients will start manifesting the typical signs of dry eye, while patients who already suffered from eye dryness will see their symptoms worsen [2, 19–23].

Most of these preparations lead to eye dryness through an anticholinergic mechanism. In fact, they compete with acetylcholine in the postsynaptic muscarinic receptor in the peripheral and/or central nervous system, thereby blocking the acetylcholine-induced stimulation of lacrimal gland and conjunctival muciparous goblet cell secretion.

#### Antidepressants and anxiolytics

Antidepressants are among the major causes of TD. In particular, tricyclic and heterocyclic antidepressants (amitriptyline, imipramine, nortriptyline, etc.) seem to be more harmful than selective serotonin reuptake inhibitors (fluoxetine, sertraline, paroxetine). It is worth reminding that these preparations are frequently used to treat anxiety and chronic pain. Therefore, they are often prescribed to patients with persistent forms of dryness, who present with anxiety and depression due to this condition.

Benzodiazepines (e.g. diazepam, alprazolam) used as anxiolytics also have anticholinergic effects, as do H_2_ receptor antagonists (e.g. cimetidine, ranitidine, famotidine) used as gastroprotectants and gastric motility regulators.

#### Antiparkinson drugs

Antiparkinson drugs, such as levodopa and pramipexole, have an anticholinergic effect, which adds up to the rare and incomplete blinking (typical in these patients) in threatening ocular surface homoeostasis and inducing TD.

#### Antihistamines

H_1_-antihistamines used for treating allergic rhinitis have an anticholinergic activity. These are often associated with decongestant vasoconstrictors, which reduce lacrimal gland blood flow. The effect of decongestants and antihistamines in the form of nasal sprays must not be underestimated, because abundant nasal blood supply causes high levels of drug absorption.

#### Antihypertensives

Negative effects on tear stability have been reported with the use of antihypertensives, such as beta blockers, which can reduce the levels of lysozyme and immunoglobulin A, as well as tear fluid production. In addition, they can produce a certain level of corneal anaesthesia, which further reduces tear production stimulation.

Even diuretics, such as hydrochlorothiazide and furosemide and triamterene, commonly used to treat hypertension, myocardial infarction and cardiac decompensation, induce eye dryness [24, 25].

#### Hormones

The use of oestrogens or oestrogen and progestin combinations (often prescribed as contraceptives or in postmenopausal hormone replacement therapies) is often associated with dry eye symptoms. The exact cause–effect correlation is still unknown; however, it is thought that there is an influence on mucus secretion and a possible link with the reduced production of tear fluid. Experimental data have shown that mucus secretion is influenced by the hormonal status even in physiological conditions [26].

Debra A. Schaumberg’s group has reported a 69% increase in dry eye symptoms in women who were taking oestrogens compared to the control group [16]. On the other hand, women who were taking progesterone alone or in combination after menopause showed a 29% increase in dry eye symptoms compared to the control group. These data highlight the significant risk of developing dry eye in oestrogen-treated patients.

Drugs used for treating overactive bladder syndrome (oxybutynin, tolterodine, fesoterodine) and antispasmodics for treating reflux syndromes and stomach disorders also have anticholinergic effects.

Furthermore, it is important to consider the effect of isotretinoin (via oral route) used in acne treatment, as it could induce meibomian gland atrophy with a negative impact on tear film stability [27, 28].

#### Polypharmacy

Polypharmacy is commonly defined as the *use of 5 or more prescription drugs*. This is a typical and growing problem in the older population. One study has shown that 12% of subjects over 60 years of age used 2 prescription drugs, 27.3%, 3 or 4, and 36.7%, 5 or more. In addition, an average of at least 2 OTC products (vitamins, aspirins, decongestants, etc.) and herbal preparation are used on a daily basis [29–32].

Although some drugs taken alone are not responsible for ocular surface damage, this may be the case when administered in combination with others, thus creating a negative synergism. Therefore, this aspect should be considered during the diagnosis. For example, it can be useful to ask the patient “are you undergoing any treatment (e.g. for hypertension, diabetes, etc.)?”, “how many and what type of drugs or supplements are you taking?”, “do you ever get dry mouth?”, “do you feel eye discomfort?”.

Polypharmacy can be a problem, because many medications interact with each other in not easily predictable ways. Sometimes, drug interaction can exceed the side effect threshold, which cannot be noticed when the drug is used alone.

Moreover, there is enough evidence that polypharmacy is a cause of dry mouth. The prevalence of dry mouth in the population that used more than 4 systemic drugs was up to 82%.

The role played by medications that can induce or worsen TD has not been analysed in-depth in targeted studies but only in incidental findings. However, despite being incomplete, these data provide enough information for drawing a few considerations:

- The role of systemic medications and systemic absorption of topical medications in inducing eye dryness is greatly underestimated;
- It is important to know whether the various drugs can have additive or even synergistic side effects;
- The role of oral polypharmacy in inducing or worsening dry eye must be analysed in-depth and taken into clinical consideration, including not only systemic prescription drugs but also topical, OTC drugs, and herbal preparations, when taking the medical history;
- The drug’s administration time may also play a role in causing symptoms of dryness.

## Patient counselling

Tear dysfunction remains an underdiagnosed and underestimated problem, despite recent publications [2].

Patients are sometimes difficult to outline because they often use a different language from doctors. On the other hand, doctors sometimes make questions that do not always help to achieve the correct diagnosis of the patient. Not to mention the little time available during an examination for questioning patients about their anamnesis, which may end up with the doctor minimising patient’s problem.

It is not uncommon for patients to be mistakenly diagnosed with TD when in fact they are suffering from other disorders. It may also happen that cases of dry eye are misdiagnosed. In other cases, despite the correct diagnosis, the patient’s satisfaction and compliance with the therapy prescribed is far than satisfactory. The causes of such problems in the management of TD are often related to a miscommunication between doctors and patients.

Many times, TD diagnosis is based on symptoms (foreign body sensation, burning, lacrimation, desire to keep the eyes closed, dryness, redness, itchiness, intermittent blurry vision, asthenopia). Unfortunately, these symptoms are not exclusive to this disease and may vary greatly from one patient to another. Moreover, there is no correlation between the symptoms reported by the patient and the doctor’s findings. For example, according to a questionnaire distributed across Europe, 22–23% of patients complained about “itchy eyes” and were mistakenly treated with an antiallergy medication [33–35].

The patient interview (medical history) is extremely important. It is essential to *give patients enough time to report all symptoms and explain their discomfort*. The symptoms reported by the patients help the doctor develop a differential diagnosis. In general, patients who feel better keeping their eyes closed are those with real dry eye.

Once the patient has reported all his/her symptoms, the doctor starts *asking specific questions to confirm the suspected disorder and exclude other causes* that are frequently mistaken for dry eye. For example, it is useful to ask a patient *which environmental conditions improve or worsen the symptoms, and investigate on the presence of other eye disorders* (allergic conjunctivitis, lacrimal duct disorders, Thygeson disease, etc.), *systemic diseases* (diabetes, rosacea, rheumatoid arthritis, thyroid problems, autoimmune diseases), *ongoing therapies* (antidepressants, gastroprotectants, hormone replacement therapy), *hormonal state* (peri-, postmenopause), *family history* (autoimmune diseases, thyroid disorders, diabetes, etc.).

Upon establishing the diagnosis of TD, it is essential to *explain to the patient very clearly what it is going on* (many patients, for example, are very worried because they associate that “heaviness” with a rise in blood pressure). In particular, the *patient must know that this is a chronic disease* that cannot be cured, and even the therapy will be chronic and adjusted over time according to the patient’s response. It is also important to tell the patient that a correct therapeutic management will allow them to return to a good quality of life, even if the therapy will not provide immediate results.

The patient must be aware that *the result will greatly depend on compliance and consistency with the therapy*. However, the patient’s compliance will depend on how much they understand the information provided by the doctor.

There is a risk of patients to end up in a three-stage vicious circle, during the first stage of which they are hopeful and think that the prescribed eye drops will solve their discomfort. After a certain period, if the treatment is not effective, they may enter the anxiety stage and think “this is getting worse… it’s going to ruin my vision… the eye doctor has underestimated my problem… the eye doctor got the diagnosis wrong”. At this point, there could be a third stage, in which patients start to look for information on Internet, talk with family and friends, and return to the ophthalmologist with all sorts of questions, worrying that they have all sorts of diseases. Often, the TD diagnosis is not convincing.

All this may justify a paradox. Patients feel more satisfied when they receive a definitive diagnosis, whatever it is. Anxious people, who do not receive a definitive diagnosis, are less likely to comply with the therapy [36]. There is a close association between depression, stress and TD in patients who have been clinically diagnosed with it or those presenting with its symptoms [37].

It is important not to underestimate the disease. For example, when patients say they have a foreign body sensation or a 24-h burning sensation, the ophthalmologist may not fully understand the problem and end up minimising it and considering it just a simple discomfort. Instead, it is important for the ophthalmologist to understand the patient’s problem and become their greatest ally to ensure an effective therapeutic strategy. It is safe to say that the level of satisfaction of both the patient and the doctor depends on two elements: the ophthalmologist must never minimise the problem and must make the patient aware that TD is a chronic disease.

It is essential to *explain to the patient that their symptoms*, including lacrimation, depend on a chronic inflammation that can get worse if not treated properly. In addition to the medical therapy, the doctor must provide *practical tips*: dry environments worsen the symptoms (heating or air conditioning); avoid direct exposure to air or heat sources; remember to close your eyes a few seconds when you read, work at a computer, or are very concentrated on an activity; humidify the environment where you work and live; hydrate; avoid using contact lenses until a healthy ocular surface is restored.

TD is a chronic pathology, and, as with all chronic conditions with no remission, the aim of the treatment is to improve the quality of life.

Therefore, it is essential to tell patients that their quality of life can improve only if they comply with the therapy provided and they leave the doctor’s office knowing that the ophthalmologist has fully understood the problem and has provided a personalised therapeutic approach. The tear substitutes must be identified also as a treatment to improve a pathological state and not just a correction of the symptoms. This is why effective and efficient communication between doctor and patient is so important.

## Identifying the problem: the 3-step method

Diagnosing TD is usually quite simple and does not involve significant differences compared to a routine eye examination.

A 3-step procedure can be a quick and useful method for a first-level diagnosis.

*Step 1*

- Ask about the symptoms (a questionnaire to be administered before the examination can be useful to prevent the patient from going into detailed and not always relevant descriptions) and their distribution in terms of intensity and frequency throughout the day;
- Observe the ocular surface, e.g. dirty tear surface, poor tear meniscus, hyperaemia restricted to the exposed interpalpebral area, dysmorphic disorder or eyelid inflammation, signs of meibomian gland dysfunction (foaming, swollen glands with purulent discharge or clogged glands, etc.), blinking rate and quality;

*Step 2*

- Stain the ocular surface:
- Instil 1 or 2% fluorescein to measure the break-up time (BUT) and to check for possible corneal and conjunctival damage. The addition of a yellow filter (Kodak Wratten Filter #12) will highlight both the tear film and the conjunctival damage (if any), which is extremely important for the diagnosis;
- Or/and use 1% lissamine green, useful for easily highlighting conjunctival and corneal damage;

*Step 3*

- Test ocular surface sensitivity and assess tear clearance in front of the ocular surface:
- Use a cotton thread, Cochet–Bonnet aesthesiometer or a more sophisticated instrument to evaluate ocular surface sensitivity and the ability of the system to react (tear secretion is the result of a stimulation);
- Re-evaluate the tear meniscus 10–15 min after staining. If the vital dye (fluorescein or lissamine green) is still present, it means that the clearance is lower than normal. This suggests a reduced tear production if the tear drainage pathway is pervious and functioning. More importantly, it indicates an increased risk of inflammation due to the surface inability to keep away toxic substances and proinflammatory cytokines.

## The impact of surgery: pre- and postoperative considerations

Anterior segment surgery is one of the major causes of ocular surface alteration. In a recent multicentre study submitted to the American Society of Cataract and Refractive Surgery, over 50% of the patients who underwent cataract surgery were diagnosed with dry eye [38, 39].

To prevent complications, it is essential to perform the surgery on an ocular surface as healthy as possible, identifying any possible risk factor (both regarding the patient and the surgery) and treating pre-existing conditions.

Ocular surface and TD can result in ocular discomfort and poor visual outcome with surgical results below expectations.

### Ocular surface dysfunction and poor visual outcome

The corneal epithelium plays an essential role in the ocular surface system (OSS) because it maintains ocular fluid homoeostasis and contributes to the tear film through the active secretion of water.

The air–tear film interface is the most important refractive surface, and the precorneal ocular surface is responsible for 2/3 of the total refractive power.

Surgery can cause tear film instability, with consequent uneven spreading across the ocular surface. This involves light deflection/scattering with the formation of a blurred image on the retina, which translates into poor visual performance.

As described in a recent publication by Baudouin et al. [40], tear film instability is one of the steps in the vicious circle of ocular surface dysfunction.

**Fig. 3:**
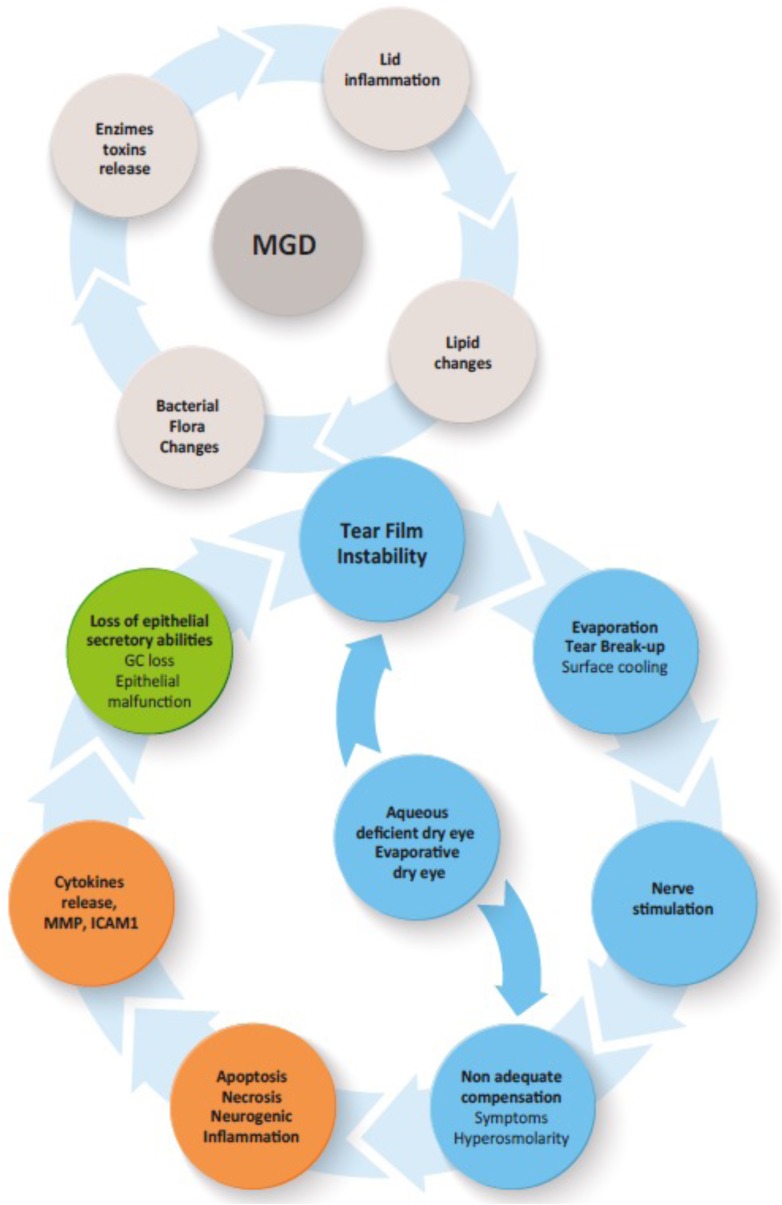
Vicious circle of the lacrimal dysfunction. *MGD* meibomian gland dysfunction, *MMP* matrix metalloproteinase, *ICAM1* intercellular adhesion molecule-1; GC loss, goblet cell loss

In particular, it involves tear hyperosmolarity, which is responsible for cell damage with apoptosis of conjunctival and corneal cells. It also triggers an inflammatory cascade, which further contributes to cell death, including mucin-secreting cells. These alterations worsen tear film instability and trigger a cycle of events that perpetuate the condition (Fig. 3).

Tear film alterations, with resulting epithelial suffering and inflammation, involve central and peripheral neuronal suffering. In particular, peripheral neuronal suffering reduces sensitivity, whereas central neuronal suffering is associated with deafferentation dysaesthesia [41].

The onset of symptoms and TD are consequences of this neuronal suffering.

There is also a characteristic timing in the onset of ocular discomfort symptoms, which get worse approximately one month after the surgery when postoperative anti-inflammatory eye drops are suspended [42].

All this is amplified if the patient has TD even before surgery. In such cases, the test scores return to their initial values approximately 3 months after surgery.

Therefore, it is essential to identify the patients who are at risk of developing this syndrome to prevent the onset of discomfort symptoms [43–45].

### Predisposing factors of chronic inflammation

There are numerous clinical conditions and iatrogenic factors that need to be evaluated before directing a patient to anterior segment surgery.

### Pre-existing clinical conditions

Predisposing conditions that may suggest the development of postoperative complications are the following: advanced age, corneal dystrophy (especially basal membrane dystrophy, which is often not diagnosed), TD, blepharitis, ocular surface inflammation even if not particularly evident, such as in the case of patients wearing contact lenses, or with pseudoexfoliation syndrome, uveitis, eyelid malpositions, use of systemic therapies that can influence tear secretion, use of topical medications that can alter the qualitative composition of the tear film, subepithelial corneal nerve plexus dysfunction and of course a pre-existing TD as well as systemic diseases, such as diabetes and rheumatologic diseases [9, 19, 46–48].

### Pharmacological factors

As for preoperative medications, it is important to consider that systemic drugs can interfere with ocular surface homoeostasis, whereas topical drugs may contain chemical preservatives (detergents), which damage the permeability of the cell membrane and the cytoplasmic content, or oxidising preservatives, which pass through the cell membrane and can interfere with cell functions [49].

At low doses, oxidising preservatives are less dangerous than detergents because the eukaryotic cells produce antioxidant enzymes [50, 51].

### Preservatives

Many pre- and postoperative eye drops contain surfactants, which alter the goblet cells and disrupt lipid layer stability. In particular, benzalkonium chloride leads to a glycocalyx alteration already 30 min after its first instillation. Its action is prolonged because the average half-life of this preservative in the conjunctiva is approximately 12 h [52]. Therefore, it is essential to use eye drops without preservatives in the preoperative prophylaxis and improve lubrication using preservative-free tear substitutes during the postoperative period.

### Surgical technique

Surgical factors (both pharmacological and those related to the surgical technique) are also important. In cataract surgery, for example, it is important to consider the epithelial toxicity of *local anaesthetics* and *disinfectants*, such as 5% povidone–iodine.

Moreover, the application of the ocular speculum can cause trauma to eyelid muscles (weakening of the orbicularis muscle) and tissues, which can lead to incomplete blinking and loss of congruence between palpebral and bulbar conjunctiva, as well as floppy eyelid syndrome. Therefore, it is essential to use non-traumatising ocular speculum and avoid “over-tensioning” eyelid opening.

The *type of incision* plays a fundamental role: it has been demonstrated that the deeper the peripheral location of the incision, the higher the risk of damaging corneal innervation. The corneal innervation can take approximately 2 years to return to preoperative levels in patients undergoing extracapsular cataract extraction. A compromised corneal innervation is responsible for an abnormal healing process, increased epithelial permeability and reduced metabolic activity. Reduced corneal sensitivity involves reduced tear secretion.

As for the construction of the *corneal tunnel*, the site and technique are crucial to avoid an uneven surface, which could cause poor distribution of the tear film in the preceding area with epithelial suffering, reduced tear film break-up time and TD. Ultrasound and microscope light exposure can also damage the ocular surface.

Similar conditions to those observed in cataract surgery occur in the postoperative period of a corneal transplant (due to the unevenness resulting from the *suture*) and in *retinal detachment* (both ab externo and ab interno), which leads to the significant remodelling of part of the conjunctiva.

Central epithelial damage is quite frequent in transplant patients due to the poor lubrication of the corneal surface, which has lost sensitivity due to innervation damage. As a result, it undergoes blinking-induced erosions, especially in the event that slightly tight sutures have flattened the surface.

The association of reduced lubrication with an inflammatory state demonstrates the importance of lubrication. In fact, TD in the form of excessive tear evaporation is the most frequently undiagnosed and underestimated cause of ocular surface inflammation. In the event of a corneal transplant, the inflammatory state of the ocular surface will result in the activation of antigen-presenting cells on the ocular surface with transplant rejection [53, 54].

In patients undergoing glaucoma filtration surgery, it is important to prevent the closure of the bleb. Moreover, dry eye can be observed in these patients, and lissamine green staining highlights areas of epithelial damage above the bleb. TD is associated with inflammation and, as a result, fibroblast recruitment. Therefore, it is essential to lubricate the ocular surface to reduce inflammation so preventing fibrosis and the formation of adherence between the conjunctiva and the sclera.

As for photorefractive surgery, both PRK and LASIK determine a significant damage of corneal nerves so inducing altered sensitivity and innervational problems. These trigger compensatory mechanisms that lead to the onset of important symptoms. Postoperative discomfort symptoms can be compared to nerve deafferentation, similar to the condition that occurs in neuropathological or neurosurgical lesions. Residual nerve fibres mediate dysaesthesia, resulting from partial peripheral lesions with loss of small afferent fibres. Pathological pain can be a problem in these patients, because the surgery causes the release of PGE_2_ and PGI_2_, which determines a sensitisation characterised by a reduced activation threshold, increased response to certain stimuli, and onset of spontaneous nociceptor activity. This sensitisation can manifest itself as hyperalgesia (i.e. an enhanced nociceptive response to certain noxious stimuli) or allodynia (i.e. sensitisation to pain in response to previously non-noxious stimuli). In these cases, the clinical picture, characterised by the typical ocular surface changes, does not justify the symptoms reported [41].

### Peri- and postoperative therapies

Today, it is known that the corneoconjunctival epithelium is not an inert tissue. In fact, it synthesises proinflammatory cytokines (interleukins and metalloproteases), or molecules that attract immunocompetent cells from the peripheral blood, thereby creating a lymphocyte homing process, which maintains the inflammatory state on the ocular surface.

Being a multifactorial condition, the preventive/therapeutic approach should be dynamic and try to identify the dominating mechanism at each follow-up to define a suitable treatment that aims at correcting the inefficient mechanism [43, 55].

In particular, the following can be very useful: eyelid hygiene in patients with meibomian gland dysfunction, avoiding the use of eye drops with vasoconstrictors, adopting (if possible before the surgery) a therapy with a tear substitute that acts on tear instability, epithelial damage and inflammation (vicious circle) and continuing it during the postoperative period [38, 44].

## Therapeutic approach: the 3+2 method

Ocular surface therapies should aim to correct the alterations of all the structures that are part of the lacrimal functional unit. A malfunction of these structures can lead to TD. Ocular surface structures are continuously exposed to the action of the external environment and therefore require efficient tear production, distribution and turnover [3, 9, 56].

Tear film, lacrimal glands (main and accessory glands, meibomian glands, goblet cells and all ocular surface secreting cells), tear drainage pathway, and corneal and conjunctival epithelium activity make up the functional unit, which protects the ocular surface by producing and maintaining an efficient tear film. The activity of the functional unit structures is regulated by the nervous system (whose endings are particularly numerous on the ocular surface) and hormones that reach it through the bloodstream.

Any agent that can alter even one of the functional unit structures can disrupt the stability of the tear film and lead to ocular surface diseases, thereby outlining the clinical picture of dry eye. This is not just the result of a tear deficiency, but a complex pathological picture, in which the tear film is unbalanced and does not provide the ocular surface with enough nourishment and protection. This alteration results in an imbalance in the production of electrolytes, proteins, and mucins, with a permanent damage to the corneal and conjunctival epithelial cells, and the activation of nerve fibres, which try to trigger the secretory activity as a compensatory mechanism. If the compensatory mechanisms activated by the functional unit structures fail to recreate a balanced system, they will trigger a series of epithelial alterations. Exposed to the noxious stimulus of a dysfunctional tear film, the epithelium will undergo the secretion of proapoptotic and proinflammatory substances, which induce a vicious circle and trigger a state of chronic suffering of the ocular surface structures. The maintenance of this vicious circle is based on three main pathogenetic factors: (1) tear film instability, (2) epithelial disease and (3) ocular surface inflammation associated or not to an innervation alteration and meibomian glands dysfunction (which contributes to tear film instability and activation of inflammation).

A therapeutic approach aiming at interrupting the vicious circle at the base of the ocular surface suffering must aim to correct the alterations of each of these aspects: this will promote the restoration of a normal homoeostasis. An appropriated therapy should be simultaneously addressed towards all these factors. The choice should be taken according to the 3+2 method (Table 5).

**Table 5:** The 3 + 2 method

Tear film instability	Inflammation	Epithelial damage
Hyaluronic Acid 0.15–0.2%	Fluid tear substitute	Trehalose
PVA 0.1%	Corticosteroids (cortisol, prednisone, etc.)	CMC 1%
HP-Guar	Cyclosporine A	Xanthan Gum
Carbopol	Omega 3	Hyaluronic Acid 0.4-0.5%
Dextran	Proinflammatory molecules inhibitors	Carbopol
etc.	etc.	etc.

A recommended therapeutic approach for the management of tear dysfunction

MGD treatment: eyelid hygiene + hot compresses + antibiotics (topic or systemic if necessary)

Innervation treatment: vitamins? Growth factors? Neurotrophic factors?

*PVA* polyvinyl alcohol, *Hp*-*guar* hydroxypropyl guar, *CMC* carboxymethylcellulose

### Address tear film instability

Tear volume, which tends to decrease with the increased evaporation, plays a primary role in tear film stabilisation. In this context, mucomimetic substances play an essential role. Among these substances, *sodium hyaluronate (HA) is nowadays considered the gold standard*.

HA is a mucopolysaccharide with a molecular weight ranging between 2 × 10^5^ and 10 × 10^6^ D. It is composed of repeating disaccharide units forming a highly negatively charged polymer, which can bind large amounts of water. This polymer has rheological properties characterised by non-Newtonian behaviour, i.e. it is provided with high viscosity, which quickly decreases when forces that determine a quick displacement are applied. This is what happens when eyelids close during a blink. This rheological property is very similar to that of normal tears. HA is present on a normal ocular surface, having been found in tears, glycocalyx, at the interface between cells and the basal membrane of the conjunctival epithelium, corneal epithelium, stroma, keratocytes, and in lacrimal gland acinar cells [57, 58]. Moreover, it has been proven that the CD44 receptor for HA is found in the cornea always closely associated with HA, thereby demonstrating the role played by this combination of molecules in modulating adhesion, growth and migration of corneal epithelial cells [57].

It has been demonstrated that the use of HA in dry eye therapies can improve the ocular surface significantly by increasing tear film stability, improving the condition of the corneoconjunctival epithelium, reducing squamous metaplasia and promoting the reappearance of muciparous goblet cells in the conjunctival epithelium [59, 60]. The HA formulation can vary and depends on the molecular weight and concentration used. The molecular weight can also depend on the origin of the molecule, i.e. from rooster comb extraction, bacterial fermentation, or chemical synthesis. The last one can reach an extremely high molecular weight, thanks to the cross-linking technique, which results in a group of molecules called hyalans [57]. The formulation of HA eye drops chosen must be based on the conditions of the ocular surface, in order to restore the normal tear film as far as possible and obtain the best possible microenvironment for the efficient repair of damaged structures [60].

### Quit ocular surface inflammation

An anti-inflammatory treatment is the second pillar of dry eye therapy. The exact pathogenesis of inflammation has not been firmly established: it could be due to the surface’s inability to keep toxic substances and proinflammatory cytokines away; a *fluid tear substitute* (not gel) with hydrophilic, mucomimetic and lubricating properties in early states of the disease is often enough to wash the ocular surface.

On the other hand, if the inflammation is clinically evident, it is probably due to an initiating stimulus that can alter ocular surface homoeostasis: environmental stress (UV-ROS, chemicals), alterations in the tear film compositions secondary to lacrimal gland inflammation, pathologic changes of neural traffic, hyperosmolarity, microtrauma from eyelids during blinking are the most recognised. These factors could play a role in inducing loss of ocular surface immunohomoeostasis and triggering Dry Eye Disease. This must be worked out in the case of evident epithelial suffering and must begin with a *corticosteroid treatment* to allow the regression, as quickly as possible, of the production of proinflammatory substances. The molecules used must have medium power and, preferably, a composition that reduces their penetration into the anterior chamber, in order to reduce, as much as possible, intraocular side effects such as increased intraocular pressure and onset of cataract. With this aim, molecules with lipophilic or fluorinated residues are to be preferred [61].

Today, corticosteroids can be associated with cyclosporine, a drug that has been recently introduced in the European market. Its immunosuppressive effect combats the activity and recruitment of T lymphocytes and inhibits apoptosis. It is important to consider that the clinical effectiveness of cyclosporine begins to manifest 2–3 months after administration. Therefore, it is important to start the anti-inflammatory treatment administering corticosteroids and cyclosporine together to obtain a rapid response to the treatment and maintain long-term results with cyclosporine alone, which is less exposed to the onset of side effects [62].

Also omega-3 fatty acids can help reduce the production of proinflammatory substances. In fact, they protect tissues from the inflammatory insult, thanks to the competitive inhibition of prostaglandin E_2_ production, and the production of metabolites known as resolvins, which block the inflammation by inhibiting immunocompetent cells [63].

### Epithelial protection

The third pillar of dry eye therapy is *the epithelium protection*. Nowadays, the conditions of the epithelial cells can be improved not only by intervening on the quality of the tear film but also by supplying substances that can protect the cells from degenerative processes, which trigger the production of proinflammatory and proapoptotic cytokines [40]. It has been demonstrated that some molecules are particularly effective in protecting the epithelium. Among these, *trehalose has drawn particular interest in ophthalmology*, as several studies have shown that it helps improve the treatment of ocular surface disorders.

This molecule is synthesised by several types of cells as a response to stress represented by extreme cold, hot, oxidation and dehydrating conditions. In particular, trehalose is synthesised by bodies as a reaction to water-deficit stress. In fact, it promotes anhydrobiosis, i.e. the ability to resist water deficit and drought. This molecule is characterised by high stability. Its behaviour when exposed to heat is particularly interesting. It first melts at 97 °C; then, with a further increase in temperature, it chrysalises. It resolidifies at 130 °C and then melts again at 203 °C.

Trehalose preserves the integrity of the cells and their intracellular organelles through multiple mechanisms, which are not yet fully known [64].

In this regard, there are three theories:

1. The vitrification theory, according to which the trehalose inside the cells creates hydrogen bonds with protein structures, thereby forming a thin hydrocolloid matrix, which protects the cells from extracellular and intracellular environmental imbalance;
2. The preferential exclusion theory, according to which trehalose interacts with water molecules, determining a redistribution with an increase in compactness and, as a result, in protein stability;
3. The water replacement theory, with the formation of hydrogen bonds that maintain the three-dimensional structure, thereby stabilising the biomolecules, which, in this way, do not undergo denaturation.

The bioprotective effect of trehalose in ocular surface alterations results from the preservation of the integrity of proteins and lipids, protection against oxidative and osmotic stress, and against hypoxia and anoxia and, consequently, from apoptosis-induced cell death.

As for the preservation of the integrity of proteins and lipids, trehalose maintains their configuration by binding with proteins. This explains the protective effect against denaturation under stress conditions, such as dehydration and frostbite.

Protection against denaturation is particularly important for the preservation of intracellular enzyme system functions, cell membrane functional properties (e.g. transport of calcium) and cellular content.

The protective effect against osmotic stress is also important for cytoprotection, as the main ocular surface alteration mechanism is frequently represented by the osmotic imbalance with the extracellular environment. Trehalose has also an antioxidant effect and its use has proven to prevent damage caused by oxygen free radicals combined with heat shock. Moreover, it increases the tolerance to anoxia because by reducing protein aggregation and maintaining their conformation, it promotes recovery after exposure to anoxic conditions.

The results of preclinical studies have shown that trehalose:

1. Hydrates and protects corneal cells from death by desiccation [64]
2. Improves resistance of ocular surface epithelium through suppression of apoptosis [65]
3. Protects the cornea from UVB-induced damage [66]
4. Prevents fibrosis and post-trabeculectomy adhesions [67].

Clinical studies based on this evidence have evaluated the efficacy of trehalose in patients with mild to severe TD compared to saline solution [68] or other tear substitutes, such as hyaluronic acid and hydroxymethylcellulose [69].

The results of these studies have shown that trehalose:

1. Improves ocular surface discomfort in patients with mild to severe TD [68]
2. Demonstrates greater efficacy in terms of improvement in subjective symptoms and objective parameters compared to tear substitutes, usually used in patients with mild to severe TD [69].

Finally, another study evaluating the corneal epithelium exposed to the toxic action of alcohol has shown that trehalose can protect epithelial cells, preserving cell membrane structures and internal organelles. This suggests that a pretreatment with trehalose can help patients who undergo photorefractive keratectomy (PRK) improving epithelial healing after surgery [70].

Meibomian glands dysfunction and innervation alteration are other two therapeutic targets to take care of.

*Meibomian gland dysfunction* is responsible for the creation of a lipid layer in the poorly efficient tear film, thereby exposing it to increased evaporation, which results in TD [71]. Modifications in the ocular surface microenvironment determine a modification in the bacterial flora, which activates innate immunity through toll-like receptors, with resulting inflammation [72]. Meibomian gland secretion is altered; therefore, it does not liquefy at the temperature of the ocular surface and remains in the excretory ducts of the glands, triggering a chronic inflammatory stimulus. The therapy is based on the use of high temperature (40-45 °C) through warm, wet compresses or eyelid-warming devices, such as Blephasteam, which allow altered lipids to melt by raising the temperature [73]. In addition to eyelid warming, the use of topical and systemic antibiotics can also help. These antibiotics have two mechanisms of action. On the one hand, they promote bacterial flora regularisation, decrease the secretion of bacterial lipases that act on altered lipid secretions and release fatty acids that are irritating to the glandular ducts. On the other hand, they have a direct anti-inflammatory effect, blocking the release of proinflammatory substances, such as metalloproteinases. The classes of antibiotics used to this end are tetracyclines (rolitetracycline, minocycline and doxycycline), administered both topically and systemically, topical macrolides (azithromycin) and topical fluoroquinolones (ofloxacin) [2, 74, 75].

The ocular surface, and especially the cornea, is the most highly innervated structure in the human body. The *innervation* plays a primary role in the homoeostasis of the functional unit, conditioning the signs and symptoms resulting from its dysfunction. In fact, a reduction in sensitivity involves hypolacrimation, resulting in the reduced production of epithelium trophic factors, such as epidermal growth factor (EGF), which is essential for epithelial trophism. In extreme cases, this epithelial suffering can lead to the formation of neurotrophic ulcers, which could seriously compromise visual function. When sensitivity is highly compromised, the clinical picture can be accompanied by mild discomfort. However, there are cases in which chronic inflammation leads to the stimulation of the nociceptors on the ocular surface, resulting in severe discomfort, which may not correspond with the clinical signs. In such cases, neuropathy can be both peripheral and central. A therapy for ocular surface innervation alterations is yet to be defined, although there are a few measures that can be taken. Again, omega-3 fatty acids can help, because one of their metabolites, neuroprotectin D, has neuroprotective properties [76, 77]. Nerve growth factor (NGF) can be useful in the event of reduced sensitivity. In fact, it has been proven to restore sensitivity and promote the healing of neurotrophic ulcers in association (if necessary) with a therapy with glycosaminoglycan (GAG) analogues, which has been recently made available in the market [78].

Therapies for ocular surface disorders must be complete, i.e. they must deal simultaneously with the alterations of the structures involved in the pathological process and be dynamic. Therefore, it is important to avoid fixed treatment regimens and adopt therapies that can change any time if the patient’s conditions require so [79]. This therapeutic approach (with progressive adjustments to the patient’s conditions) allows for the improvement and stabilisation of the patient’s clinical conditions and an overall improvement in their quality of life.

The treatment for ocular disorder should take into consideration the use of formulations without preservatives [the most commonly used preservative is the benzalkonium chloride (BAK)] [80].

Patients with dry eye are at particular risk; in fact, the low tear volume allows higher concentrations of BAK to remain in contact with the cornea for longer periods of time [81].

Long-term use of preservative-containing artificial tears is associated with an increased risk of adverse events and epithelial surface damage and diminished compliance due to ocular irritation [50].

Experimental studies in cultured conjunctival cells have shown increased cytotoxic effects of BAK in hyperosmolarity conditions with characteristic cell death process, including caspase-dependent and caspase-independent apoptosis and oxidative stress [51]. This suggests that BAK administered in an eye already submitted to hyperosmolar conditions would be more toxic than in a healthy normal ocular surface.

That is why *preservative*-*free formulation is absolutely necessary* as well as therapies for ocular surface disorders and for patients with lacrimal dysfunction.

## Conclusions

TD is highly prevalent in the population aged over 45 years and consists of the imbalance of the ocular surface functional unit. Its pathogenesis is multifactorial, and the clinical picture varies greatly, ranging from very mild to virtually invalidating conditions with a severe impact on the quality of life. In the last few years, an increasing interest arouse, due to the difficulties in diagnosis and treatment. The most recent researches have shown that the ocular surface epithelium is the main target of the disease as a consequence of an altered and non-functional tear film. The epithelium will promote inflammation and apoptosis, through the production of proinflammatory and proapoptotic molecules as the consequence of the insult. A nervous impairment and the establishment of a vicious cycle, determining the chronicity of the process, will follow.

**Fig. 4:**
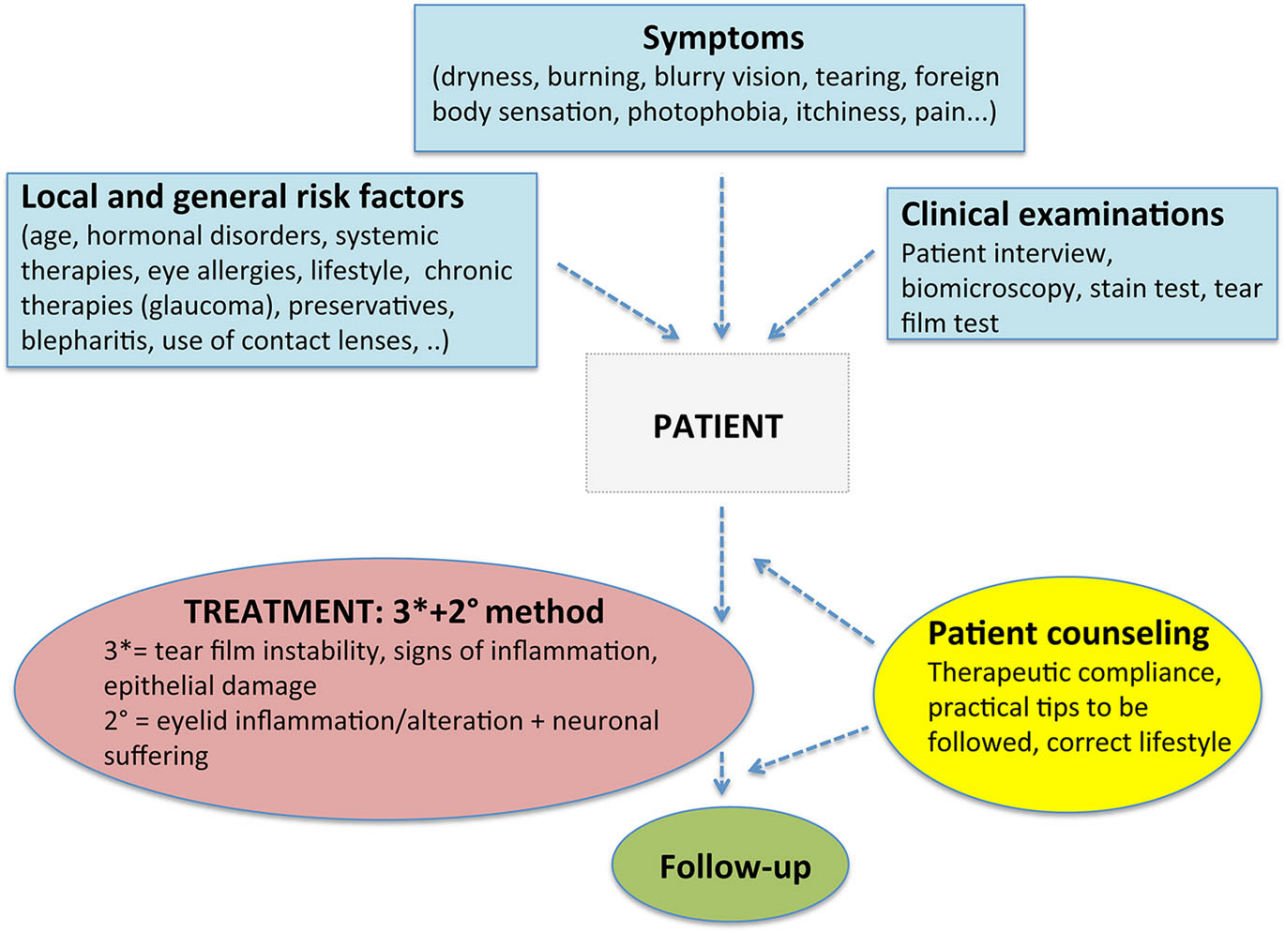
Multi-item flowchart to better define the diagnosis and assess the better treatment in patients with tear dysfunction

The need for a clear identification of the different involvement of each component of the ocular surface structure, in order to assess the best treatment possible, has prompted us to identify a useful approach to diagnose and treat TD (Fig. 4). The main aspect to be considered is that, being dry eye a multifactorial disease with a multiform presentation and course, the treatment should be adapted to the patient’s condition rather than be just a standard treatment. Using the simple flowchart illustrated in Fig. 4 will guide from the right diagnosis to the best treatment possible.
